# A6 PROTEASE-ACTIVATED RECEPTOR (PAR)-2 ACTIVATION ENHANCES EPITHELIAL WOUND HEALING MIGRATION THROUGH SRC AND RAC1 PATHWAYS

**DOI:** 10.1093/jcag/gwac036.006

**Published:** 2023-03-07

**Authors:** L Lucena Perico, W K MacNaughton

**Affiliations:** Physiology and Pharmacology, University of Calgary, Calgary, Canada

## Abstract

**Background:**

Protease-activated receptors (PARs) and their activating enzymes have been postulated to play a role in inflammatory bowel disease (IBD) pathogenesis, but the specific roles of PAR2 in disease initiation and progression remain unclear. PAR2 activation has both pro-proliferative and pro-migratory effects and could be involved with the restoration of the epithelial barrier following injury. We previously showed that PAR2 activation increases epithelial wound healing through ERK, PI3K, and JNK pathways. However, the role of Src kinase and RhoGTPases, which PAR2 also activates, is not known.

**Purpose:**

We hypothesized that PAR-2 activation induces wound healing in intestinal epithelial cells through Src and Rac1 activity.

**Method:**

Circular wounds were made in T84 colonic epithelial cell monolayers. Wounded monolayers were treated with the PAR2 activating peptide, 2-furoyl-LIGRLO (2fLI, 5 μM), or the inactive control reverse-sequence peptide, 2-furoyl-OLRGIL (2fO, 5 μM), and live-cell imaging was used to record wound healing over a 24-hr period. Proliferation and apoptosis were measured using EdU and TUNEL assays, respectively. The mechanism of action was evaluated using inhibitors of Src (PP2), EGFR (PD153035), MLCK (ML-7), ROCK (Y-27632), Rac1 (NSC 23766), and Cdc42 (ML141), and western blot (WB) was used to confirm the protein levels of p-Src (Y416), p-ERK1/2, p-JNK, and p-PI3K. For immunofluorescence, images of E-cadherin and F-actin were taken to capture the entire wound border and surrounding cells.

**Result(s):**

PAR2 activation by 2fLI promoted wound healing compared to 2fO or vehicle control at the 24-hr time point (*p*<0.05). PAR2 activation had no effect on proliferation at the wound edge and did not affect apoptosis but did enhance lamellipodia/filopodia formation (*p*<0.001). These findings indicate that PAR2 reprograms the cells toward a migratory rather than a proliferative phenotype. When we investigated the mechanisms of action, the Src tyrosine kinase inhibitor, PP2, blocked PAR2-induced wound healing (*p*<0.0001). PAR2 activation increased Src phosphorylation (Y416, *p*<0.05) and the immunofluorescence showed a rise in actin cable formation at the wound edge and a reduction in lamellipodia/filopodia formation in the group treated with PP2 when compared to 2fLI (*p*<0.001). Although PAR2 activation increases the phosphorylation of ERK1/2 and JNK, this did not happen through Src. Furthermore, PAR2 did not increase the phosphorylation of PI3K as confirmed by WB analysis. Inhibition of EGFR (PD153035), MLCK (ML-7), ROCK (Y-27632), and Cdc42 (ML-141) did not alter PAR2-induced wound healing (*p*>0.05). In contrast, Rac1 inhibition by NSC23766 completely abrogated the PAR2-induced wound healing (*p*<0.05).

**Conclusion(s):**

PAR2 activation drives wound healing via Src tyrosine kinase and Rac1 activities. These findings provide a further mechanism whereby PAR2 can participate in the resolution of intestinal wounds in gastrointestinal inflammatory diseases.

**Disclosure of Interest:**

None Declared

